# Supplementary material for: Fdo1, Fkh1, Fkh2, and the Swi6–Mbp1 MBF complex regulate Mcd1 levels to impact eco1 rad61 cell growth in Saccharomyces cerevisiae
Source: Genetics. 2024 Aug 7;228(2):iyae128. doi: 10.1093/genetics/iyae128 (PMC11457938; doi:10.1093/genetics/iyae128)
Supplement: iyae128_Supplementary_Data [file iyae128_supplementary_data.zip › Figures_S1-S4_GENETICS-2024-307170/File S3/Supplemental Figure Legends.docx]

**Supplemental Figure Legends**

**Figure S1. *MCD1* overexpression in *eco1Δ rad61Δ fkh1Δ* triple mutant cells.**

Growth of ten-fold serial dilutions of *eco1Δ rad61Δ* double mutant cells overexpressing *MCD1*, compared to *eco1Δ rad61Δ fkh1Δ* triple mutant cells overexpressing vector alone, or *MCD1* (2 independent isolates shown). Temperature and days of growth are indicated.

**Figure S2. PCR identification of *FKH1* or *FKH2* single deletions in *eco1Δ rad61Δ kanMX6 cells*.**

PCR analyses of genomic DNAs obtained from *eco1Δ rad61Δ kanMX6* (*kanMX6* replacing *FKH1* and *FKH2*) spores from YBS4450 dissections. Schematics at top and bottom highlight PCR strategies used to identify *FKH1:kanMX6* (*fkh1Δ*) and *FKH2:kanMX6* (*fkh1Δ*), respectively. PCR products from 13 spores (lanes marked 1-13), along with a no-DNA control (N) are shown. Molecular weight (MW) marker bands of 1371, 1264 and 702 nucleotides also shown.

**Figure S3. PCR identification of *FKH1* and *FKH2* co-deletions in *eco1Δ rad61Δ kanMX6 ECO1::LEU2* cells.**

PCR analyses of genomic DNAs obtained from *eco1Δ rad61Δ kanMX6* (*kanMX6* replacing *FKH1* and *FKH2*) *ECO1::LEU2* spores from YBS4450 dissections. Schematics at top and bottom highlight PCR strategies used to identify *FKH1:kanMX6* (*fkh1Δ*) and *FKH2:kanMX6* (*fkh1Δ*), respectively. PCR products from 6 spores (lanes marked 1-13), along with a no-DNA control (N) are shown. Molecular weight (MW) marker bands of 1371, 1264 and 702 nucleotides also shown.

**Figure S4. PCR identification of *FKH1* and *FKH2* co-deletions in *eco1Δ rad61Δ kanMX6 2µm-TRP1-MCD1* cells.**

PCR analyses of genomic DNAs obtained from *eco1Δ rad61Δ kanMX6* (*kanMX6* replacing *FKH1* and *FKH2*) *2µ-TRP1-MCD1* spores from YBS4435 dissections. PCR strategies used are identical to that shown in Figures S3 and S3. PCR products from 2 spores are shown. No-DNA control (N) and PCR products indicative of *FKH1* (*fkh1Δ*) and *FKH2* (*fkh2Δ*) deletions are shown. Molecular weight (MW) marker bands of 1371, 1264 and 702 nucleotides also shown.
