# Supplementary material for: Fdo1, Fkh1, Fkh2, and the Swi6–Mbp1 MBF complex regulate Mcd1 levels to impact eco1 rad61 cell growth in Saccharomyces cerevisiae
Source: Genetics. 2024 Aug 7;228(2):iyae128. doi: 10.1093/genetics/iyae128 (PMC11457938; doi:10.1093/genetics/iyae128)
Supplement: iyae128_Supplementary_Data [file iyae128_supplementary_data.zip › Table_S1_GENETICS-2024-307170/File S1/Table S1.docx]

**Supplemental Table 1:** Strains used in this study.

**YPH499** (Sikorski and Hieter, 1989)

*MATa ura3-52 lys2-801_amber ade2-101_ochre trp1-Δ63 his3-Δ200 leu2-Δ1*

**YBS255** (Skibbens et al., 1999)

*MATa ECO1/ECO1:LEU2 eco1/eco1::HIS3 ade2-101 his3∆200 leu2∆1 lys2-801 trp1∆63 ura3-53*

**YBS4450** (Current Study)

*MATa/alpha eco1::HIS3/eco1::HIS3 rad61::URA3/rad61::URA3 fkh1::kanMX6 fkh2::kanMX6 ECO1:LEU2*

**YBS4535** (Current Study)

*MATa/alpha ECO1::HIS3/ECO1::HIS3 rad61::URA3/rad61::URA3 fkh1::kanMX6 fkh2::kanMX6 ECO1:LEU2 (2µm-TRP1-MCD1)*

**YBS4547** (Current Study)

*MATalpha eco1::HIS3 rad61::URA3 fkh2::kanMX6 (2µm-TRP1-MCD1)*

**YBS4548** (Current Study)

*MATalpha eco1::HIS3 rad61::URA3 fkh1::kanMX6 fkh2::kanMX6 (2µm-TRP1-MCD1)*

**YMM828** (Maradeo and Skibbens, 2010)

*MATalpha eco1::HIS3 rad61::URA3 ade2-101 his3∆200 leu2∆1 lys2-801 trp1∆63 ura3-52*

**YMM829** (Maradeo and Skibbens, 2010)

*MATa eco1::HIS3 rad61::URA3 ade2-101 his3∆200 leu2∆1 lys2-801 trp1∆63 ura3-52*

**YGS4** (Current Study)

*MATalpha eco1::HIS3 rad61::URA3 fdo1::KAN ade2-101 his3∆200 leu2∆1 lys2- 801 trp1∆63 ura3-52 (isolate 2)*

**YGS36** (Current Study)

*MATalpha eco1::HIS3 rad61::URA3 fdo1::KAN ade2-101 his3∆200 leu2∆1 lys2-801 trp1∆63 ura3-52 (isolate 8a)*

**YGS37** (Current Study)

*MATalpha eco1::HIS3 rad61::URA3 fdo1::KAN ade2-101 his3∆200 leu2∆1 lys2-801 trp1∆63 ura3-52 (isolate 8e)*

**YGS1** (Current Study)

*MATalpha eco1::HIS3 rad61::URA3 fdo1::KAN ade2-101 his3∆200 leu2∆1 lys2-801 trp1∆63 ura3-52*

**YGS3** (Current Study)

*MATalpha eco1::HIS3 rad61::URA3 fdo1::KAN ade2-101 his3∆200 leu2∆1 lys2-801 trp1∆63 ura3-52*

**YGS5** (Current Study)

*MATa eco1::HIS3 rad61::URA3 fdo^250-1029Δ^::KAN ade2-101 his3∆200 leu2∆1 lys2-801 trp1∆63 ura3-52 (isolate IV)*

**YGS6** (Current Study)

*MATalpha eco1::HIS3 rad61::URA3 fdo^250-1029Δ^::KAN ade2-101 his3∆200 leu2∆1 lys2-801 trp1∆63 ura3-52 (isolate V)*

**YGS11** (Current Study)

*MATalpha eco1::HIS3 rad61::URA3 fkh1::KAN ade2-101 his3∆200 leu2∆1 lys2-801 trp1∆63 ura3-52 (isolate 5a)*

**YGS12** (Current Study)

*MATalpha eco1::HIS3 rad61::URA3 fkh1::KAN ade2-101 his3∆200 leu2∆1 lys2-801 trp1∆63 ura3-52 (isolate 5b)*

**YGS13** (Current Study)

*MATalpha eco1::HIS3 rad61::URA3 fkh2:: KAN ade2-101 his3∆200 leu2∆1 lys2-801 trp1∆63 ura3-52 (isolate 7a)*

**YGS14** (Current Study)

*MATalpha eco1::HIS3 rad61::URA3 fkh2:: KAN ade2-101 his3∆200 leu2∆1 lys2-801 trp1∆63 ura3-52 (isolate 7b)*

**YGS26** (Current Study)

*MATa ECO1:LEU2 eco1::HIS3 ade2-101 his3∆200 leu2∆1 lys2-801 trp1∆63 ura3-53 pRS424 (isolate 1b)*

**YGS28** (Current Study)

*MATalpha eco1::HIS3 rad61::URA3 ade2-101 his3∆200 leu2∆1 lys2-801 trp1∆63 ura3-52 pRS 424 (isolate 2b)*

**YSG80** (Current Study)

*MATalpha eco1::HIS3 rad61::URA3 ade2-101 his3∆200 leu2∆1 lys2-801 trp1∆63 ura3-52 pGS7 (isolate 4a)*

**YGS84** (Current Study)

*MATalpha eco1::HIS3 rad61::URA3 ade2-101 his3∆200 leu2∆1 lys2-801 trp1∆63 ura3-52 pGS8 (isolate 6a)*

**YGS53** (Current Study)

*MATalpha eco1::HIS3 rad61::URA3 ade2-101 his3∆200 leu2∆1 lys2-801 trp1∆63 ura3-52 pGS6 (isolate b)*

**YGS55** (Current Study)

*MATalpha eco1::HIS3 rad61::URA3 ade2-101 his3∆200 leu2∆1 lys2-801 trp1∆63 ura3-52 pGS6 (isolate d)*

**YGS79** (Current Study)

*MATa ECO1:LEU2 eco1::HIS3 ade2-101 his3∆200 leu2∆1 lys2-801 trp1∆63 ura3-53 pGS7 (isolate 3a)*

**YGS83** (Current Study)

*MATa ECO1:LEU2 eco1::HIS3 ade2-101 his3∆200 leu2∆1 lys2-801 trp1∆63 ura3-53 pGS8 (isolate 5a)*

**YGS126** (Current Study)

*MATa ECO1:LEU2 eco1::HIS3 ade2-101 his3∆200 leu2∆1 lys2-801 trp1∆63 ura3-53 pGS6 (isolate A)*

**YGS127** (Current Study)

*MATa ECO1:LEU2 eco1::HIS3 ade2-101 his3∆200 leu2∆1 lys2-801 trp1∆63 ura3-53 pGS6 (isolate B)*

**YGS99** (Current Study)

*MATalpha eco1::HIS3 rad61::URA3 ade2-101 his3∆200 leu2∆1 lys2-801 trp1∆63 ura3-52 pGS11 (isolate 4a)*

**YGS101** (Current Study)

*MATalpha eco1::HIS3 rad61::URA3 ade2-101 his3∆200 leu2∆1 lys2-801 trp1∆63 ura3-52 pGS12 (isolate 6a)*

**YGS103** (Current Study)

*MATalpha eco1::HIS3 rad61::URA3 ade2-101 his3∆200 leu2∆1 lys2-801 trp1∆63 ura3-52 pGS13 (isolate 8a)*

**YGS98** (Current Study)

*MATa ECO1:LEU2 eco1::HIS3 ade2-101 his3∆200 leu2∆1 lys2-801 trp1∆63 ura3-53 pGS11 (isolate 3a)*

**YGS100** (Current Study)

*MATa ECO1:LEU2 eco1::HIS3 ade2-101 his3∆200 leu2∆1 lys2-801 trp1∆63 ura3-53 pGS12 (isolate 5a)*

**YGS102** (Current Study)

*MATa ECO1:LEU2 eco1::HIS3 ade2-101 his3∆200 leu2∆1 lys2-801 trp1∆63 ura3-53 pGS13 (isolate 7a)*

**YGS27** (Current Study)

*MATa ECO1:LEU2 eco1::HIS3 ade2-101 his3∆200 leu2∆1 lys2-801 trp1∆63 ura3-53 pBS1476 (isolate 3b)*

**YGS29** (Current Study)

*MATalpha eco1::HIS3 rad61::URA3 ade2-101 his3∆200 leu2∆1 lys2-801 trp1∆63 ura3-52 pBS1476 (isolate 4b)*

**YGS30** (Current Study)

*MATalpha eco1::HIS3 rad61::URA3 ade2-101 his3∆200 leu2∆1 lys2-801 trp1∆63 ura3-52 pBS1476 (isolate 4c)*

**YGS105** (Current Study)

*MATalpha eco1::HIS3 rad61::URA3 ade2-101 his3∆200 leu2∆1 lys2-801 trp1∆63 ura3-52 pGS14 (isolate 4a)*

**YGS107** (Current Study)

*MATalpha eco1::HIS3 rad61::URA3 ade2-101 his3∆200 leu2∆1 lys2-801 trp1∆63 ura3-52 pGS15 (isolate 6a)*

**YGS109** (Current Study)

*MATalpha eco1::HIS3 rad61::URA3 ade2-101 his3∆200 leu2∆1 lys2-801 trp1∆63 ura3-52 pGS16 (isolate 8b)*

**YGS104** (Current Study)

*MATa ECO1:LEU2 eco1::HIS3 ade2-101 his3∆200 leu2∆1 lys2-801 trp1∆63 ura3-53 pGS14 (isolate 3a)*

**YGS106** (Current Study)

*MATa ECO1:LEU2 eco1::HIS3 ade2-101 his3∆200 leu2∆1 lys2-801 trp1∆63 ura3-53 pGS15 (isolate 5a)*

**YGS108** (Current Study)

*MATa ECO1:LEU2 eco1::HIS3 ade2-101 his3∆200 leu2∆1 lys2-801 trp1∆63 ura3-53 pGS16 (isolate 7a)*

**YGS158** (Current Study)

*MATa ura3-52 lys2-801_amber ade2-101_ochre trp1-Δ63 his3-Δ200 leu2-Δ1 pRS425 (isolate 1a)*

**YGS160** (Current Study)

*MATalpha eco1::HIS3 rad61::URA3 ade2-101 his3∆200 leu2∆1 lys2-801 trp1∆63 ura3-52 pRS425 (isolate 2a)*

**YGS163** (Current Study)

*MATalpha eco1::HIS3 rad61::URA3 ade2-101 his3∆200 leu2∆1 lys2-801 trp1∆63 ura3-52 pGS31 (isolate 4a)*

**YSG165** (Current Study)

*MATalpha eco1::HIS3 rad61::URA3 ade2-101 his3∆200 leu2∆1 lys2-801 trp1∆63 ura3-52 pGS32 (isolate 6a)*

**YGS167** (Current Study)

*MATalpha eco1::HIS3 rad61::URA3 ade2-101 his3∆200 leu2∆1 lys2-801 trp1∆63 ura3-52 pGS33 (isolate 8a)*

**YGS 162** (Current Study)

*MATa ura3-52 lys2-801_amber ade2-101_ochre trp1-Δ63 his3-Δ200 leu2-Δ1 pGS31 (isolate 3a)*

**YGS 164** (Current Study)

*MATa ura3-52 lys2-801_amber ade2-101_ochre trp1-Δ63 his3-Δ200 leu2-Δ1 pGS32 (isolate 5a)*

**YGS 166** (Current Study)

*MATa ura3-52 lys2-801_amber ade2-101_ochre trp1-Δ63 his3-Δ200 leu2-Δ1 pGS33 (isolate 7a)*

**YGS 168** (Current Study)

*MATa ura3-52 lys2-801_amber ade2-101_ochre trp1-Δ63 his3-Δ200 leu2-Δ1 pRS424, pRS425 (isolate 9a)*

**YGS 130** (Current Study)

*MATalpha eco1::HIS3 rad61::URA3 ade2-101 his3∆200 leu2∆1 lys2-801 trp1∆63 ura3-52 pRS424, pRS425 (isolate 10a)*

**YGS134** (Current Study)

*MATalpha eco1::HIS3 rad61::URA3 ade2-101 his3∆200 leu2∆1 lys2-801 trp1∆63 ura3-52 pGS31, pGS14 (isolate 12a)*

**YGS138** (Current Study)

*MATalpha eco1::HIS3 rad61::URA3 ade2-101 his3∆200 leu2∆1 lys2-801 trp1∆63 ura3-52 pGS32, pGS14 (isolate 14a)*

**YGS142** (Current Study)

*MATalpha eco1::HIS3 rad61::URA3 ade2-101 his3∆200 leu2∆1 lys2-801 trp1∆63 ura3-52 pGS33, pGS14 (isolate 16a)*

**YGS170** (Current Study)

*MATa ura3-52 lys2-801_amber ade2-101_ochre trp1-Δ63 his3-Δ200 leu2-Δ1 pGS31, pGS14 (isolate 10a)*

**YGS171** (Current Study)

*MATa ura3-52 lys2-801_amber ade2-101_ochre trp1-Δ63 his3-Δ200 leu2-Δ1 pGS32, pGS14 (isolate 11a)*

**YGS172** (Current Study)

*MATa ura3-52 lys2-801_amber ade2-101_ochre trp1-Δ63 his3-Δ200 leu2-Δ1 pGS33, pGS 14 (isolate 12a)*

**YGS179** (Current Study)

*MATalpha eco1::HIS3 rad61::URA3 ade2-101 his3∆200 leu2∆1 lys2-801 trp1∆63 ura3-52 pGS35 (isolate 1a)*

**YGS193** (Current Study)

*MATalpha eco1::HIS3 rad61::URA3 ade2-101 his3∆200 leu2∆1 lys2-801 trp1∆63 ura3-52 pGS11, pBS1018 (isolate 1a)*

**YGS195** (Current Study)

*MATalpha eco1::HIS3 rad61::URA3 ade2-101 his3∆200 leu2∆1 lys2-801 trp1∆63 ura3-52 pGS11, pGS35 (isolate 2a)*

**YGS196** (Current Study)

*MATalpha eco1::HIS3 rad61::URA3 ade2-101 his3∆200 leu2∆1 lys2-801 trp1∆63 ura3-52 pGS11, pGS35 (isolate 2b)*

**YGS176** (Current Study)

*MATalpha eco1::HIS3 rad61::URA3 fkh2:: KAN ade2-101 his3∆200 leu2∆1 lys2-801 trp1∆63 ura3-52 pBS1017 (isolate 3a)*

**YGS177** (Current Study)

*MATalpha eco1::HIS3 rad61::URA3 fkh2:: KAN ade2-101 his3∆200 leu2∆1 lys2-801 trp1∆63 ura3-52 pBS1476 (isolate 7a)*

**YGS178**

*MATalpha eco1::HIS3 rad61::URA3 fkh2:: KAN ade2-101 his3∆200 leu2∆1 lys2-801 trp1∆63 ura3-52 pBS1476 (isolate 8a)*

**YGS180** (Current Study)

*MATalpha eco1::HIS3 rad61::URA3 ade2-101 his3∆200 leu2∆1 lys2-801 trp1∆63 ura3-52 pGS7, pBS1018 (isolate 2a)*

**YGS181** (Current Study)

*MATalpha eco1::HIS3 rad61::URA3 ade2-101 his3∆200 leu2∆1 lys2-801 trp1∆63 ura3-52 pGS7, pGS35 (isolate 3a)*

**YGS182** (Current Study)

*MATalpha eco1::HIS3 rad61::URA3 ade2-101 his3∆200 leu2∆1 lys2-801 trp1∆63 ura3-52 pGS7, pGS35 (isolate 3b)*

**YGS183** (Current Study)

*MATalpha eco1::HIS3 rad61::URA3 ade2-101 his3∆200 leu2∆1 lys2-801 trp1∆63 ura3-52 pGS6, pBS1018 (isolate 5a)*

**YGS184** (Current Study)

*MATalpha eco1::HIS3 rad61::URA3 ade2-101 his3∆200 leu2∆1 lys2-801 trp1∆63 ura3-52 pGS6, pGS35 (isolate 6a)*

**YGS185** (Current Study)

*MATalpha eco1::HIS3 rad61::URA3 ade2-101 his3∆200 leu2∆1 lys2-801 trp1∆63 ura3-52 pGS6, pGS35 (isolate 6b)*

**YGS197** (Current Study)

*MATalpha eco1::HIS3 rad61::URA3 ade2-101 his3∆200 leu2∆1 lys2-801 trp1∆63 ura3-52 pGS14, pBS1018 (isolate 1a)*

**YGS199** (Current Study)

*MATalpha eco1::HIS3 rad61::URA3 ade2-101 his3∆200 leu2∆1 lys2-801 trp1∆63 ura3-52 pGS14, pGS35 (isolate 2a)*

**YGS200** (Current Study)

*MATalpha eco1::HIS3 rad61::URA3 ade2-101 his3∆200 leu2∆1 lys2-801 trp1∆63 ura3-52 pGS14, pGS35 (isolate 2b)*

**YGS173** (Current Study)

*MATalpha eco1::HIS3 rad61::URA3 fkh1::KAN ade2-101 his3∆200 leu2∆1 lys2-801 trp1∆63 ura3-52 pBS1017 (isolate 1a)*

**YGS174** (Current Study)

*MATalpha eco1::HIS3 rad61::URA3 fkh1::KAN ade2-101 his3∆200 leu2∆1 lys2-801 trp1∆63 ura3-52 pBS1476 (isolate 5a)*

**YGS175** (Current Study)

*MATalpha eco1::HIS3 rad61::URA3 fkh1::KAN ade2-101 his3∆200 leu2∆1 lys2-801 trp1∆63 ura3-52 pBS1476 (isolate 6a)*
