# Supplementary material for: Fdo1, Fkh1, Fkh2, and the Swi6–Mbp1 MBF complex regulate Mcd1 levels to impact eco1 rad61 cell growth in Saccharomyces cerevisiae
Source: Genetics. 2024 Aug 7;228(2):iyae128. doi: 10.1093/genetics/iyae128 (PMC11457938; doi:10.1093/genetics/iyae128)
Supplement: iyae128_Supplementary_Data [file iyae128_supplementary_data.zip › Table_S2_GENETICS-2024-307170/File S2/Table S2.docx]

**Supplemental Table 2:** DNA oligos sequences used in this study.

**oRVS23**

5' GAT TGT CGC ACC TGA TTG CC 3'

**oRVS3204**

5’ CGT ATT TGC TTT ATT TTT AAG GAC AAA TTT AAA AGT CAT ACA GCA CGG ATC CCC GGG TTA ATT AA 3’

**oRVS3206**

5’ TCA TCT TCA CAA GAG ACA CTT AGT GTC GAT AGA GAG ATT GAT GAA CGG ATC CCC GGG TTA ATT AA 3’

**oRVS3205**

5’ GTG TAT TTC TTG CCG TCA AGA ACA AAC ATT ATC TAG TAT CTT TGA GAA TTC GAG CTC GTT TAA AC 3’

**oRVS3207**

5’ GCC TAG CGG TGG AAT GCG GGC 3’

**oRVS3222**

5’ AGT GTG TAA ATT GTG CGT TCA ATT AGC AAA GAA AGG CTT GGA GAG ACA CAG TAA TAA TAA CGG ATC CCC GGG TTA ATT AA 3’

**oRVS3223**

5’ CTT AAC GGG TCT TTG TTC TTT ATT GTT TAA TAA TAC ATA TGG GTT CGA CGA CGC TGA ATT GAA TTC GAG CTC GTT TAA AC 3’

**oRVS3224**

5’ GTC ATG TCC AGG TAG ACA AAC 3’

**oRVS3225**

5’ ACA TAA ATA TTG GTG TGC TCC CTC CGT TTC CTT TAT TGA AAC TTT ATC AAT GCG CAA GAA CGG ATC CCC GGG TTA ATT AA 3’

**oRVS3226**

5’ TGC AGC TTA GCC ATT TCT CAT TCA TTT CTT TAG TCT TAG TGA TTC ACC TTG TTT CTT GTC GAA TTC GAG CTC GTT TAA AC 3’

**oRVS3227**

5’ CCC TGT GCC ATT TGG ATT TTC 3’

**oRVS3304**

5’ AATT CCGCGG ATT ACC AAA AAA AAA AAA AAG 3’

**oRVS3305**

5’ ATAT CTCGAG TCA AAA AAT TCA TTC AAT AAA 3’

**oRVS3292**

5’ AATT CCGCGG TAG GTA AAT TAG TAA ATA AAG 3’

**oRVS3293**

5’ ATAT CTCGAG TAG AAT GAA AGG AAA GCA GCT 3’

**oRVS3306**

5’ AATT CCGCGG TTT CTA TAT AAA TTA CGC TAT 3’

**oRVS3307**

5’ ATAT CTCGAG TTT AGA TAA ACC ATC ATC AAA 3’

**oRVS3308**

5’ AATT CCGCGG CAT ATA ACT GAA GAT AGT ATC 3’

**oRVS3309**

5’ ATAT CCCGGG TTT ATT CCA TCG GAT ATA TGA 3’

**oRVS3312**

5’ AATT CCGCGG TAT TTC TGA TCG TGA TTA AAC 3’

**oRVS3313**

5’ ATAT CTCGAG AGC ACT GCT TAC TGT TAT GTC 3’
