# Supplementary figures and images for: Fdo1, Fkh1, Fkh2, and the Swi6–Mbp1 MBF complex regulate Mcd1 levels to impact eco1 rad61 cell growth in Saccharomyces cerevisiae
Source: Genetics. 2024 Aug 7;228(2):iyae128. doi: 10.1093/genetics/iyae128 (PMC11457938; doi:10.1093/genetics/iyae128)

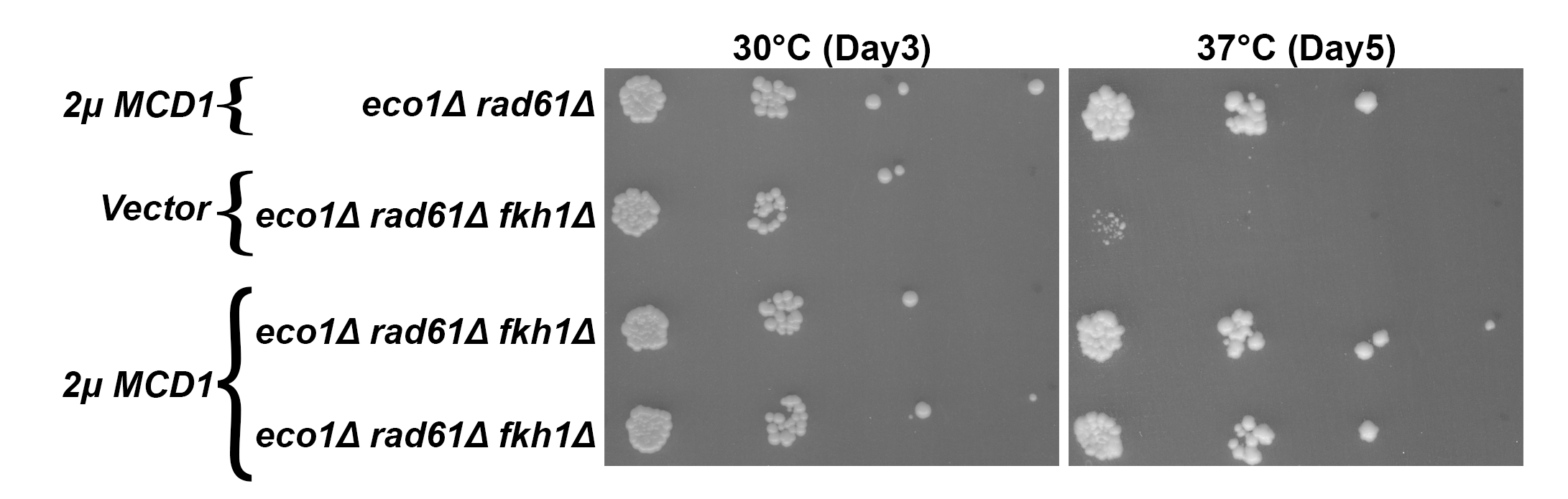

Supplement: iyae128_Supplementary_Data [file iyae128_supplementary_data.zip › Figures_S1-S4_GENETICS-2024-307170/File S3/Figure S1.tif]

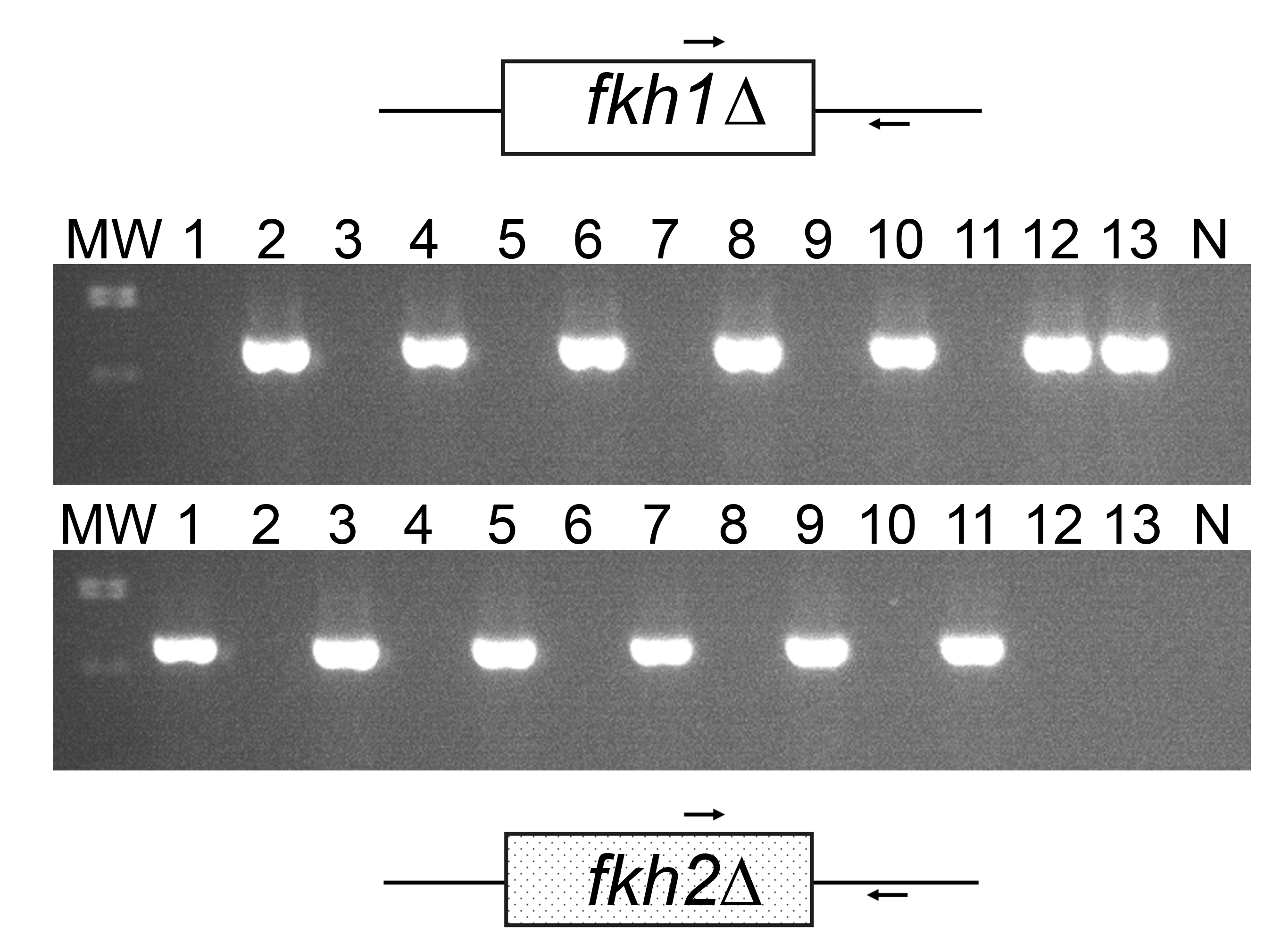

Supplement: iyae128_Supplementary_Data [file iyae128_supplementary_data.zip › Figures_S1-S4_GENETICS-2024-307170/File S3/Figure S2.tif]

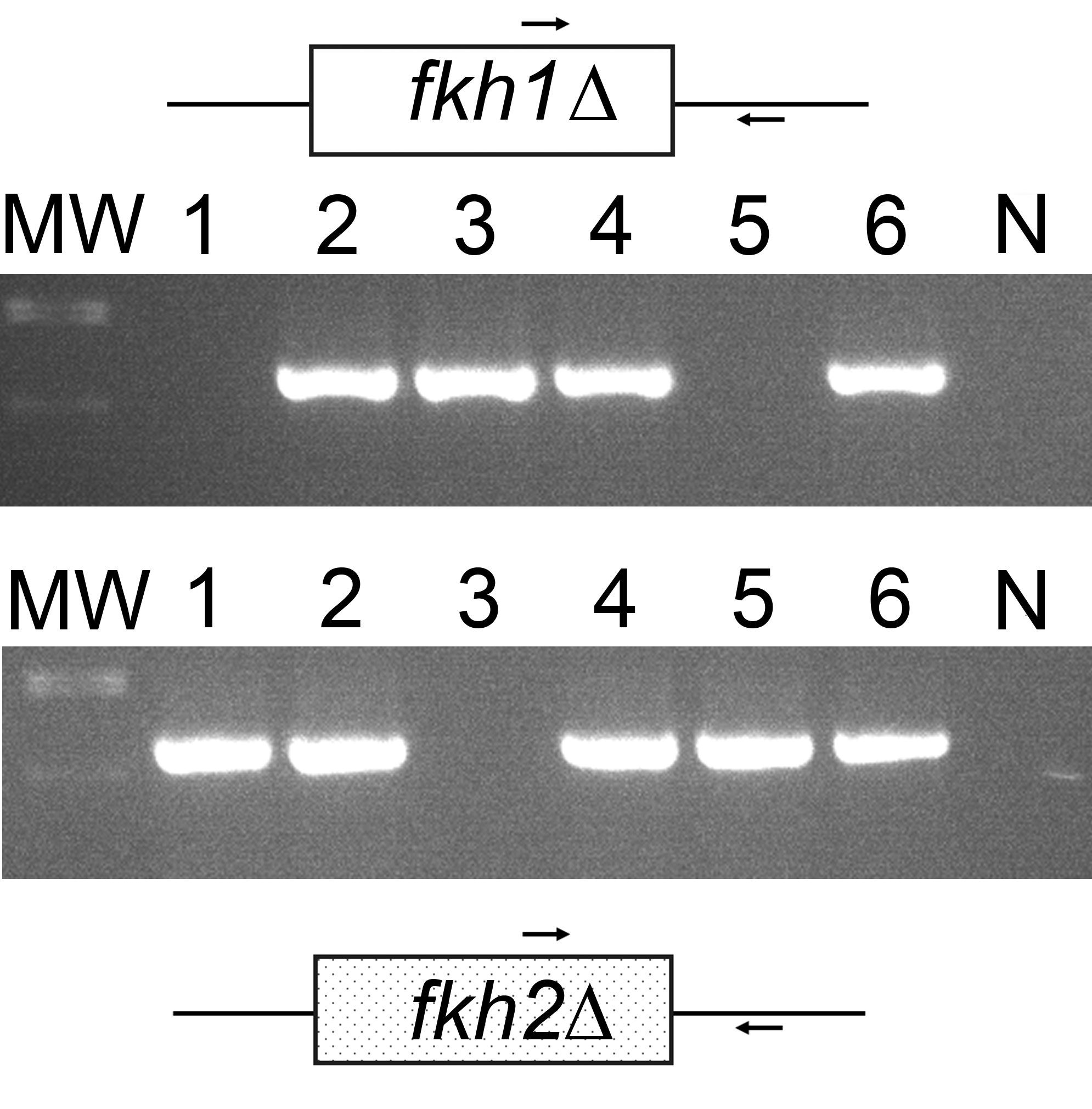

Supplement: iyae128_Supplementary_Data [file iyae128_supplementary_data.zip › Figures_S1-S4_GENETICS-2024-307170/File S3/Figure S3.tif]

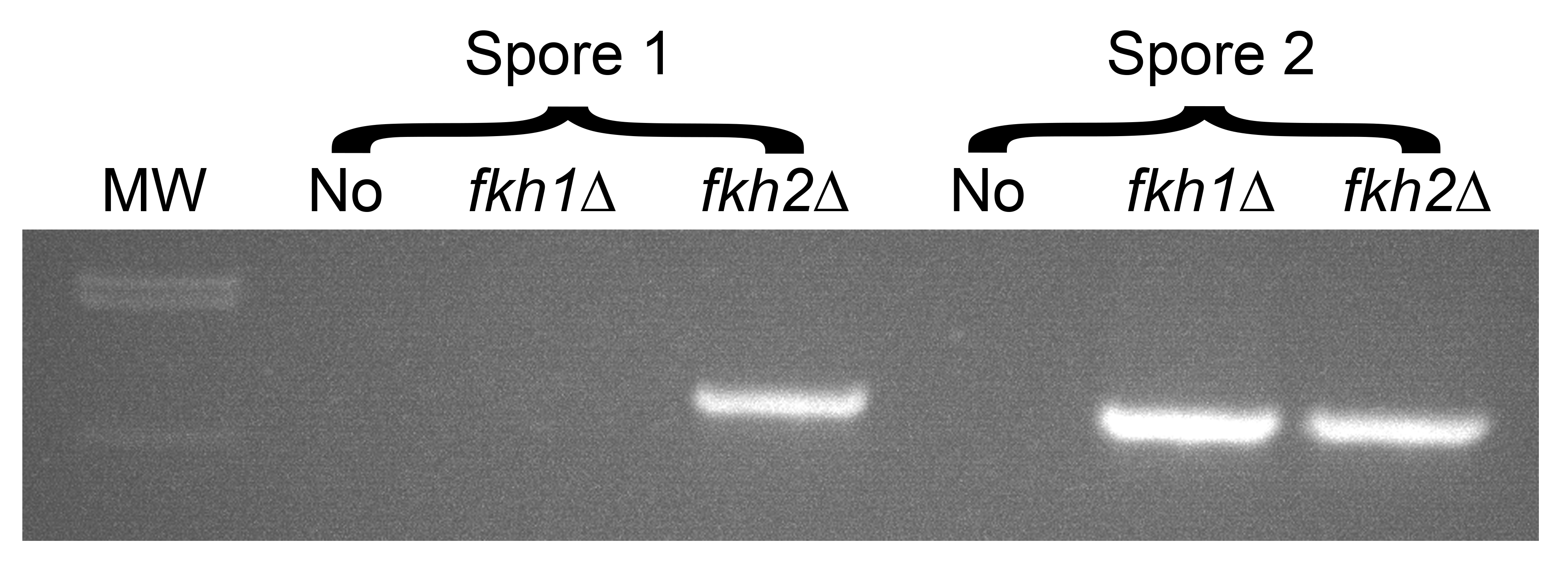

Supplement: iyae128_Supplementary_Data [file iyae128_supplementary_data.zip › Figures_S1-S4_GENETICS-2024-307170/File S3/Figure S4.tif]
